# 2-(3-Oxo-3,4-dihydro-2*H*-1,4-benzothia­zin-4-yl)acetamide

**DOI:** 10.1107/S1600536810036305

**Published:** 2010-09-15

**Authors:** Azher Saeed, Zaid Mahmood, Shiyao Yang, Muhammad Salim, Muhammad Saleem Akhtar

**Affiliations:** a Institute of Chemistry, University of the Punjab, Lahore 54590, Pakistan; b Department of Chemistry, College of Chemistry and Chemical Engineering, Xiamen University, Xiamen 361005, People’s Republic of China; c Department of Chemistry, Govt. Islamia College, Civil Lines, Lahore, Pakistan

## Abstract

In the title compound, C_10_H_10_N_2_O_2_S, the thia­zine ring approximates to an envelope form with the S atom in the flap position. The amide group attached to the acetate group is almost perpendicular to the mean plane of the thia­zine ring [dihedral angle = 88.83 (8)°]. In the crystal, inversion dimers linked by pairs of N—H⋯O hydrogen bonds occur. Further N—H⋯O and C—H⋯O hydrogen bonds link the dimers into a three-dimensional network.

## Related literature

For a related structure and background references, see: Saeed *et al.* (2010 ▶). For graph-set notation, see: Bernstein *et al.* (1995 ▶)
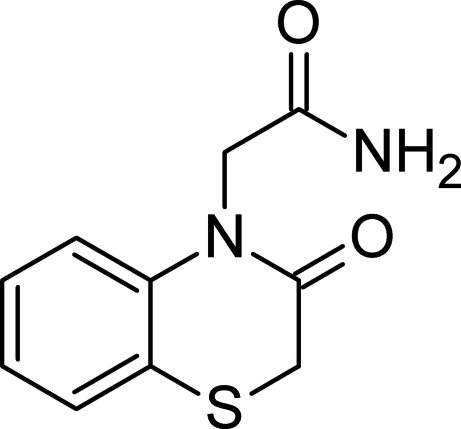


## Experimental

### 

#### Crystal data

C_10_H_10_N_2_O_2_S
*M* *_r_* = 222.26
Monoclinic, 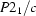

*a* = 8.0652 (6) Å
*b* = 4.8415 (3) Å
*c* = 26.1517 (19) Å
β = 94.798 (4)°
*V* = 1017.58 (12) Å^3^
*Z* = 4
Mo *K*α radiation
μ = 0.30 mm^−1^
*T* = 296 K
0.28 × 0.09 × 0.06 mm

#### Data collection

Bruker Kappa APEXII CCD diffractometer
Absorption correction: multi-scan (*SADABS*; Bruker, 2007 ▶) *T*_min_ = 0.921, *T*_max_ = 0.982
11611 measured reflections
2544 independent reflections
1693 reflections with *I* > 2σ(*I*)
*R*_int_ = 0.039

#### Refinement

*R*[*F* ^2^ > 2σ(*F* ^2^)] = 0.039
*wR*(*F* ^2^) = 0.107
*S* = 1.02
2544 reflections
142 parameters
H atoms treated by a mixture of independent and constrained refinement
Δρ_max_ = 0.24 e Å^−3^
Δρ_min_ = −0.26 e Å^−3^

### 

Data collection: *APEX2* (Bruker, 2007 ▶); cell refinement: *SAINT* (Bruker, 2007 ▶); data reduction: *SAINT*; program(s) used to solve structure: *SHELXS97* (Sheldrick, 2008 ▶); program(s) used to refine structure: *SHELXL97* (Sheldrick, 2008 ▶); molecular graphics: *ORTEPII* (Johnson, 1976 ▶); software used to prepare material for publication: *SHELXL97*.

## Supplementary Material

Crystal structure: contains datablocks I, global. DOI: 10.1107/S1600536810036305/hb5628sup1.cif

Structure factors: contains datablocks I. DOI: 10.1107/S1600536810036305/hb5628Isup2.hkl

Additional supplementary materials:  crystallographic information; 3D view; checkCIF report

## Figures and Tables

**Table 1:** Hydrogen-bond geometry (Å, °)

*D*—H⋯*A*	*D*—H	H⋯*A*	*D*⋯*A*	*D*—H⋯*A*
N2—H1*N*⋯O1^i^	0.87 (3)	2.18 (3)	3.026 (2)	164 (2)
N2—H2*N*⋯O2^ii^	0.84 (3)	2.04 (3)	2.873 (2)	174 (2)
C8—H8*B*⋯O1^iii^	0.97	2.57	3.532 (2)	173

Symmetry codes: (i) 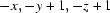; (ii) 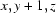; (iii) 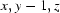.

**Fig. 1.**
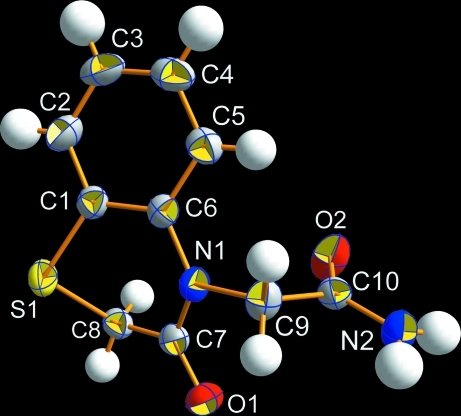
The molecular structure of (I) with displacement ellipsoids drawn at the 50% probability level.

**Fig. 2.**
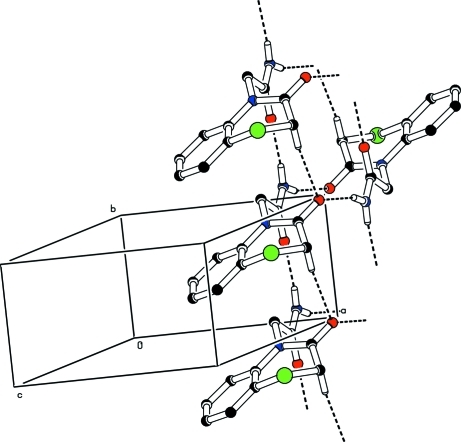
The crystal packing of (I) with intermolecular hydrogen bonds shown by dashed lines. The hydrogen atom not involved in hydrogen bonding have been omitted for clarity.

## supplementary crystallographic information

### Comment

As part of our ongoing studies of 1,4-thiazine compounds (Saeed *et al.*, 2010) we have synthesized 2-(3-oxo-2,3-dihydro benzo[b][1,4]thiazin-4-yl)acetamide for derivaziation and we report here the structure of the title compound.

The bond lengths and bond angles of the structure of the title compound is in comparison with our previously published structure of 2-(3-Oxo-3,4-dihydro-2*H*-1,4-benzothiazin-4-yl)acetohydrazide (II) (Saeed *et al.*, 2010). These molecules only differ in amide (I) and hydrazide (II) groups attached to carbonyl carbon of acetate. The dihedral angle between the two rings C1–C6 and C1/C6/N1/C7/C8/S1 are almost same in these molecules i.e. 17.47 (0.09)° and 16.77 (0.10)° respectively. The amide group C9/C10/O2/N2 attached to the thiazine ring is oriented at dihedral angle of 72.05 (0.08)° and 88.83 (0.08)° with respect to the aromatic and thiazine ring. The amido hydrogens atoms are involved N–H···O type interactions with the oxygens of two different molecules. The N–H···O and weak C–H···O form dimers which results in 16 members ring motif *R*_2_^2^(16) (Bernstein *et al.*, 1995) along the b axes.

### Refinement

The C-H H-atoms were positioned gemetrically with C—H = 0.93 Å for aromatic and C—H = 0.97 Å for the methylene carbon atoms and were refined using a riding model with *U*_iso_(H) = 1.2 *U*_eq_(C). The N-H H atoms were located in difference map with N—H= 0.84 (4)–0.87 (3) Å, *U*_iso_(H) = 1.2 for N atoms.

### Crystal data

**Table:**

C_10_H_10_N_2_O_2_S	*F*(000) = 464
*M**_r_* = 222.26	*D*_x_ = 1.451 Mg m^−^^3^
Monoclinic, *P*2_1_/*c*	Mo *K*α radiation, λ = 0.71073 Å
Hall symbol: -P 2ybc	Cell parameters from 2429 reflections
*a* = 8.0652 (6) Å	θ = 2.5–24.3°
*b* = 4.8415 (3) Å	µ = 0.30 mm^−^^1^
*c* = 26.1517 (19) Å	*T* = 296 K
β = 94.798 (4)°	Needle, colorless
*V* = 1017.58 (12) Å^3^	0.28 × 0.09 × 0.06 mm
*Z* = 4	

### Data collection

**Table:**

Bruker Kappa APEXII CCD diffractometer	2544 independent reflections
Radiation source: fine-focus sealed tube	1693 reflections with *I* > 2σ(*I*)
graphite	*R*_int_ = 0.039
φ and ω scans	θ_max_ = 28.4°, θ_min_ = 1.6°
Absorption correction: multi-scan (*SADABS*; Bruker, 2007)	*h* = −10→10
*T*_min_ = 0.921, *T*_max_ = 0.982	*k* = −6→6
11611 measured reflections	*l* = −34→34

### Refinement

**Table:**

Refinement on *F*^2^	Primary atom site location: structure-invariant direct methods
Least-squares matrix: full	Secondary atom site location: difference Fourier map
*R*[*F*^2^ > 2σ(*F*^2^)] = 0.039	Hydrogen site location: inferred from neighbouring sites
*wR*(*F*^2^) = 0.107	H atoms treated by a mixture of independent and constrained refinement
*S* = 1.02	*w* = 1/[σ^2^(*F*_o_^2^) + (0.0425*P*)^2^ + 0.2819*P*] where *P* = (*F*_o_^2^ + 2*F*_c_^2^)/3
2544 reflections	(Δ/σ)_max_ < 0.001
142 parameters	Δρ_max_ = 0.24 e Å^−^^3^
0 restraints	Δρ_min_ = −0.26 e Å^−^^3^

### Special details

**Table:**

Experimental. To a solution of (1.56 g)ethyl 2-(3-oxo-2,3-dihydrobenzo[b][1,4]thiazin-4-yl)- acetate in 10.0 ml ethanol, 5.0 ml of 33% ammonia was added and the mixture was left for a week at room temperature. The crystals of 2-(3-oxo-2,3-dihydrobenzo[1,4]thiazin-4-yl)acetamide appeared were filtered, washed with water and dried.(M.p 475k)
Geometry. All s.u.'s (except the s.u. in the dihedral angle between two l.s. planes) are estimated using the full covariance matrix. The cell s.u.'s are taken into account individually in the estimation of s.u.'s in distances, angles and torsion angles; correlations between s.u.'s in cell parameters are only used when they are defined by crystal symmetry. An approximate (isotropic) treatment of cell s.u.'s is used for estimating s.u.'s involving l.s. planes.
Refinement. Refinement of F^2^ against ALL reflections. The weighted R-factor wR and goodness of fit S are based on F^2^, conventional R-factors R are based on F, with F set to zero for negative F^2^. The threshold expression of F^2^ > 2σ(F^2^) is used only for calculating R-factors(gt) etc. and is not relevant to the choice of reflections for refinement. R-factors based on F^2^ are statistically about twice as large as those based on F, and R- factors based on ALL data will be even larger.

### Fractional atomic coordinates and isotropic or equivalent isotropic displacement parameters (Å^2^)

**Table:**

	*x*	*y*	*z*	*U*_iso_*/*U*_eq_	
C1	0.3288 (2)	0.0539 (4)	0.31875 (7)	0.0388 (4)	
C2	0.4502 (3)	−0.1224 (4)	0.30259 (9)	0.0529 (5)	
H2	0.4358	−0.2023	0.2702	0.063*	
C3	0.5914 (3)	−0.1799 (5)	0.33408 (10)	0.0584 (6)	
H3	0.6726	−0.2962	0.3228	0.070*	
C4	0.6121 (3)	−0.0650 (5)	0.38215 (9)	0.0530 (6)	
H4	0.7083	−0.1011	0.4032	0.064*	
C5	0.4909 (2)	0.1035 (4)	0.39939 (8)	0.0437 (5)	
H5	0.5047	0.1759	0.4324	0.052*	
C6	0.3480 (2)	0.1667 (3)	0.36793 (7)	0.0338 (4)	
C7	0.0611 (2)	0.3388 (4)	0.36763 (7)	0.0378 (4)	
C8	0.0096 (2)	0.1364 (4)	0.32606 (7)	0.0416 (4)	
H8A	−0.1005	0.1838	0.3108	0.050*	
H8B	0.0043	−0.0472	0.3407	0.050*	
C9	0.2668 (3)	0.5317 (4)	0.42842 (7)	0.0419 (5)	
H9A	0.2076	0.7047	0.4225	0.050*	
H9B	0.3851	0.5713	0.4306	0.050*	
C10	0.2227 (2)	0.4090 (3)	0.47892 (7)	0.0364 (4)	
N1	0.22507 (19)	0.3468 (3)	0.38519 (6)	0.0361 (4)	
N2	0.2048 (2)	0.5907 (4)	0.51556 (7)	0.0455 (4)	
O1	−0.04093 (19)	0.4905 (3)	0.38558 (6)	0.0541 (4)	
O2	0.2099 (2)	0.1598 (3)	0.48455 (6)	0.0621 (5)	
S1	0.15349 (7)	0.13660 (13)	0.277323 (19)	0.05234 (19)	
H1N	0.170 (3)	0.539 (5)	0.5448 (10)	0.063*	
H2N	0.200 (3)	0.758 (5)	0.5079 (9)	0.063*	

### Atomic displacement parameters (Å^2^)

**Table:**

	*U*^11^	*U*^22^	*U*^33^	*U*^12^	*U*^13^	*U*^23^
C1	0.0372 (11)	0.0433 (10)	0.0364 (10)	−0.0020 (8)	0.0064 (8)	0.0017 (8)
C2	0.0504 (14)	0.0582 (13)	0.0518 (13)	0.0043 (11)	0.0148 (11)	−0.0085 (10)
C3	0.0427 (13)	0.0595 (13)	0.0757 (17)	0.0117 (11)	0.0204 (12)	0.0056 (12)
C4	0.0333 (12)	0.0619 (13)	0.0638 (14)	0.0005 (10)	0.0038 (10)	0.0158 (11)
C5	0.0363 (11)	0.0515 (11)	0.0430 (11)	−0.0067 (9)	0.0009 (9)	0.0047 (9)
C6	0.0341 (10)	0.0320 (8)	0.0360 (9)	−0.0051 (7)	0.0064 (8)	0.0037 (7)
C7	0.0421 (11)	0.0369 (9)	0.0354 (9)	0.0030 (8)	0.0097 (8)	0.0096 (8)
C8	0.0337 (10)	0.0518 (11)	0.0389 (10)	0.0008 (9)	0.0000 (8)	0.0045 (9)
C9	0.0567 (13)	0.0299 (9)	0.0395 (10)	−0.0068 (9)	0.0072 (9)	−0.0014 (8)
C10	0.0422 (11)	0.0288 (9)	0.0378 (10)	0.0007 (8)	0.0015 (8)	0.0012 (7)
N1	0.0407 (9)	0.0342 (8)	0.0339 (8)	−0.0020 (7)	0.0052 (7)	−0.0014 (6)
N2	0.0665 (13)	0.0331 (8)	0.0372 (9)	−0.0014 (8)	0.0064 (9)	0.0003 (7)
O1	0.0539 (10)	0.0549 (8)	0.0553 (9)	0.0169 (7)	0.0163 (8)	0.0037 (7)
O2	0.1082 (14)	0.0280 (7)	0.0531 (9)	−0.0020 (7)	0.0254 (9)	0.0033 (6)
S1	0.0471 (3)	0.0784 (4)	0.0311 (3)	0.0047 (3)	0.0010 (2)	−0.0017 (2)

### Geometric parameters (Å, °)

**Table:**

C1—C2	1.391 (3)	C7—N1	1.364 (2)
C1—C6	1.394 (3)	C7—C8	1.496 (3)
C1—S1	1.754 (2)	C8—S1	1.794 (2)
C2—C3	1.377 (3)	C8—H8A	0.9700
C2—H2	0.9300	C8—H8B	0.9700
C3—C4	1.372 (3)	C9—N1	1.459 (2)
C3—H3	0.9300	C9—C10	1.517 (3)
C4—C5	1.377 (3)	C9—H9A	0.9700
C4—H4	0.9300	C9—H9B	0.9700
C5—C6	1.393 (3)	C10—O2	1.221 (2)
C5—H5	0.9300	C10—N2	1.318 (2)
C6—N1	1.422 (2)	N2—H1N	0.87 (3)
C7—O1	1.225 (2)	N2—H2N	0.84 (3)
			
C2—C1—C6	119.62 (19)	C7—C8—H8A	109.4
C2—C1—S1	120.25 (16)	S1—C8—H8A	109.4
C6—C1—S1	120.14 (15)	C7—C8—H8B	109.4
C3—C2—C1	120.7 (2)	S1—C8—H8B	109.4
C3—C2—H2	119.7	H8A—C8—H8B	108.0
C1—C2—H2	119.7	N1—C9—C10	112.25 (14)
C4—C3—C2	119.8 (2)	N1—C9—H9A	109.2
C4—C3—H3	120.1	C10—C9—H9A	109.2
C2—C3—H3	120.1	N1—C9—H9B	109.2
C3—C4—C5	120.3 (2)	C10—C9—H9B	109.2
C3—C4—H4	119.8	H9A—C9—H9B	107.9
C5—C4—H4	119.8	O2—C10—N2	123.82 (18)
C4—C5—C6	120.8 (2)	O2—C10—C9	121.34 (17)
C4—C5—H5	119.6	N2—C10—C9	114.82 (15)
C6—C5—H5	119.6	C7—N1—C6	123.91 (15)
C5—C6—C1	118.77 (17)	C7—N1—C9	115.64 (16)
C5—C6—N1	120.77 (17)	C6—N1—C9	120.03 (16)
C1—C6—N1	120.44 (17)	C10—N2—H1N	120.5 (16)
O1—C7—N1	121.15 (18)	C10—N2—H2N	118.6 (16)
O1—C7—C8	121.10 (19)	H1N—N2—H2N	119 (2)
N1—C7—C8	117.75 (16)	C1—S1—C8	95.57 (9)
C7—C8—S1	111.06 (13)		
			
C6—C1—C2—C3	−2.0 (3)	N1—C9—C10—N2	−158.55 (18)
S1—C1—C2—C3	177.41 (17)	O1—C7—N1—C6	176.58 (16)
C1—C2—C3—C4	0.8 (3)	C8—C7—N1—C6	−2.8 (2)
C2—C3—C4—C5	1.2 (3)	O1—C7—N1—C9	4.0 (2)
C3—C4—C5—C6	−1.9 (3)	C8—C7—N1—C9	−175.37 (15)
C4—C5—C6—C1	0.6 (3)	C5—C6—N1—C7	−153.56 (17)
C4—C5—C6—N1	−178.24 (17)	C1—C6—N1—C7	27.6 (2)
C2—C1—C6—C5	1.3 (3)	C5—C6—N1—C9	18.7 (2)
S1—C1—C6—C5	−178.14 (14)	C1—C6—N1—C9	−160.18 (16)
C2—C1—C6—N1	−179.83 (17)	C10—C9—N1—C7	78.0 (2)
S1—C1—C6—N1	0.7 (2)	C10—C9—N1—C6	−94.8 (2)
O1—C7—C8—S1	136.66 (16)	C2—C1—S1—C8	142.29 (17)
N1—C7—C8—S1	−43.93 (19)	C6—C1—S1—C8	−38.29 (16)
N1—C9—C10—O2	23.3 (3)	C7—C8—S1—C1	57.82 (15)

### Hydrogen-bond geometry (Å, °)

**Table:**

*D*—H···*A*	*D*—H	H···*A*	*D*···*A*	*D*—H···*A*
N2—H1N···O1^i^	0.87 (3)	2.18 (3)	3.026 (2)	164 (2)
N2—H2N···O2^ii^	0.84 (3)	2.04 (3)	2.873 (2)	174 (2)
C8—H8B···O1^iii^	0.97	2.57	3.532 (2)	173

Symmetry codes: (i) −*x*, −*y*+1, −*z*+1; (ii) *x*, *y*+1, *z*; (iii) *x*, *y*−1, *z*.
